# Supplementary material for: A UMLS-based spell checker for natural language processing in vaccine safety
Source: BMC Med Inform Decis Mak. 2007 Feb 12;7:3. doi: 10.1186/1472-6947-7-3 (PMC1805499; doi:10.1186/1472-6947-7-3)
Supplement: Additional file 13 — RAP application directory. Sets up directory for RAP (RDF application for PHP) source code [file 1472-6947-7-3-S13.gz › rap/doc/phpdoc/__filesource/fsource_ontModel__ontModelIndividual.php.html]

xml version="1.0" encoding="iso-8859-1"?


File Source for Individual.php


# Source for file Individual.php

Documentation is available at Individual.php

```
1. <?PHP
2. /**
3. * ----------------------------------------------------------------------------------
4. * Class: Individual
5. * ----------------------------------------------------------------------------------
6. *
7. * @package     ontModel
8. */


11. /**
12. * Interface that encapsulates an individual in an ontology, sometimes referred
13. * to as a fact or assertion.
14. * In order to be recognised as an individual, rather than a generic resource,
15. * at least one rdf:type statement, referring to a known class, must be present
16. * in the model.
17. *
18. * <BR><BR>History:
19. * <LI>10-05-2004                : First version of this class.</LI>
20. *
21. * @version  V0.9.1
22. * @author Daniel Westphal <mail at d-westphal dot de>
23. *
24. *
25. * @package     ontModel
26. * @access    public
27. ***/

29. class Individual extends OntResource
30. {

32. /**
33. * Constructor
34. * You can supply a uri
35. *
36. * @param string $uri
37. * @access    public
38. */
39. function Individual($uri = null)
40. {
41. parent::OntResource($uri);
42. }
43. }
44. ?>
```

Documentation generated on Thu, 7 Jul 2005 13:41:28 +0200 by phpDocumentor 1.3.0RC3
